# Insulin-like growth factor 1 in heat stress-induced neuroinflammation: novel perspective about the neuroprotective role of chromium

**DOI:** 10.1007/s44154-023-00105-1

**Published:** 2023-07-12

**Authors:** Songlin Wang, Kanghui Hou, Siqi Gui, Yue Ma, Shuai Wang, Shanting Zhao, Xiaoyan Zhu

**Affiliations:** College of Veterinary Medicine, Northwest A&F University, Yangling, 712100 China

**Keywords:** IGF-1, Chromium, Heat stress, Neuroinflammation, Hsp70

## Abstract

Heat stress (HS) can cause a series of stress responses, resulting in numerous negative effects on the body, such as the diminished food intake, carcass quality and reproductive capacity. In addition to the negative effects on the peripheral system, HS leads to central nervous system (CNS) disorders given its toll on neuroinflammation. This neuroinflammatory process is mainly mediated by microglia and astrocytes, which are involved in the activation of glial cells and the secretion of cytokines. While the regulation of inflammatory signaling has a close relationship with the expression of heat shock protein 70 (Hsp70), HS-induced neuroinflammation is closely related to the activation of the TLR4/NF-κB pathway. Moreover, oxidative stress and endoplasmic reticulum (ER) stress are key players in the development of neuroinflammation. Chromium (Cr) has been widely shown to have neuroprotective effects in both humans and animals, despite the lack of mechanistic evidence. Evidence has shown that Cr supplementation can increase the levels of insulin-like growth factor 1 (IGF-1), a major neurotrophic factor with anti-inflammatory and antioxidant effects. This review highlights recent advances in the attenuating effects and potential mechanisms of Cr-mediated IGF-1 actions on HS-induced neuroinflammation, providing presently existing evidence supporting the neuroprotective role of Cr.

## Introduction

In recent years, the rise in global temperature has posed great challenges to the health of humans and animals, with heat-related diseases gradually attracting the attention of researchers. Of note, high temperature environments cause great economic losses to animal husbandry production in many regions (Ebi et al. 2021; Goel 2021).

Heat stress (HS) represents the sum of systemic, non-specific reactions in animals present in high-temperature and high-humidity environments caused by insufficient heat dissipation and uncontrolled thermoregulation (Roenfeldt 1998). Under HS conditions, a series of pathophysiological responses, including high temperature, reduction of feed intake, dehydration, dyspnea, increased heart rate and gastrointestinal injuries, may emerge prominently (Vargas and Marino 2016; Chen et al. 2021a, b). Notably, due to the activation of the hypothalamic–pituitary–adrenal (HPA) axis and increased production of glucocorticoids, HS causes an inhibitory effect on the immune system (Bagath et al. 2019). In addition, HS is detrimental to the reproductive capacity and product quality of animals. In fact, it may cause bull lower semen quality (Morrell 2020), decreased milk of dairy cows (Tao et al. 2020) and egg production of laying hens (Li et al. 2020), and the development of pale, soft and exudative (PSE) meat in acute HS or dark, firm and dry (DFD) meat in chronic HS in pigs (Gonzalez-Rivas et al. 2020).

Moreover, in the central nervous system (CNS), HS can lead to many neurological disorders due to its highly inflammatory conditions. Neuroinflammation refers to the inflammation in the CNS (especially brain tissue), which can be triggered by noxious stimuli and conditions, including HS (Medzhitov 2008; Lee et al. 2015; Chauhan et al. 2017; Zhao et al. 2021a, b). Acute inflammation may have a protective effect on animals (e.g., providing protection against infections or promoting tissue repair and angiogenesis) (Aggarwal et al. 2006; Varin and Gordon 2009). However, when inflammation becomes chronic, it is highly detrimental to brain tissue, often causing synaptic dysfunction, inhibition of neurogenesis, neuronal death and cognitive impairment (Lyman et al. 2014). 

Chromium (Cr) is a promising agent against the adverse effects of HS in animals (Bin-Jumah et al. 2020). Cr acts as a second messenger and amplifies insulin signals, which facilitates the role of insulin neuromodulation (Vincent 2015; Nakabeppu 2019). Evidence suggests that Cr has positive effects on the brain, namely improving cognitive function in the elderly and ameliorating depression (Krikorian et al. 2010; Andrieux et al. 2021). Importantly, the anti-inflammatory and antioxidant effects as well as the modulation of insulin-like growth factor 1 (IGF-1) signaling of Cr supplements are well-recognized (Peng et al. 2010; Chen et al. 2014; Ullah Khan et al. 2014; Morvaridzadeh et al. 2022). Therefore, in this article, we addressed the mechanisms underlying HS-induced neuroinflammation and explored the link between Cr and IGF-1 signaling, further unraveling the potential role of Cr-mediated IGF-1 in inhibiting neuroinflammation induced by HS.

## HS effects on the CNS and neuroinflammation

**Fig. 1:**
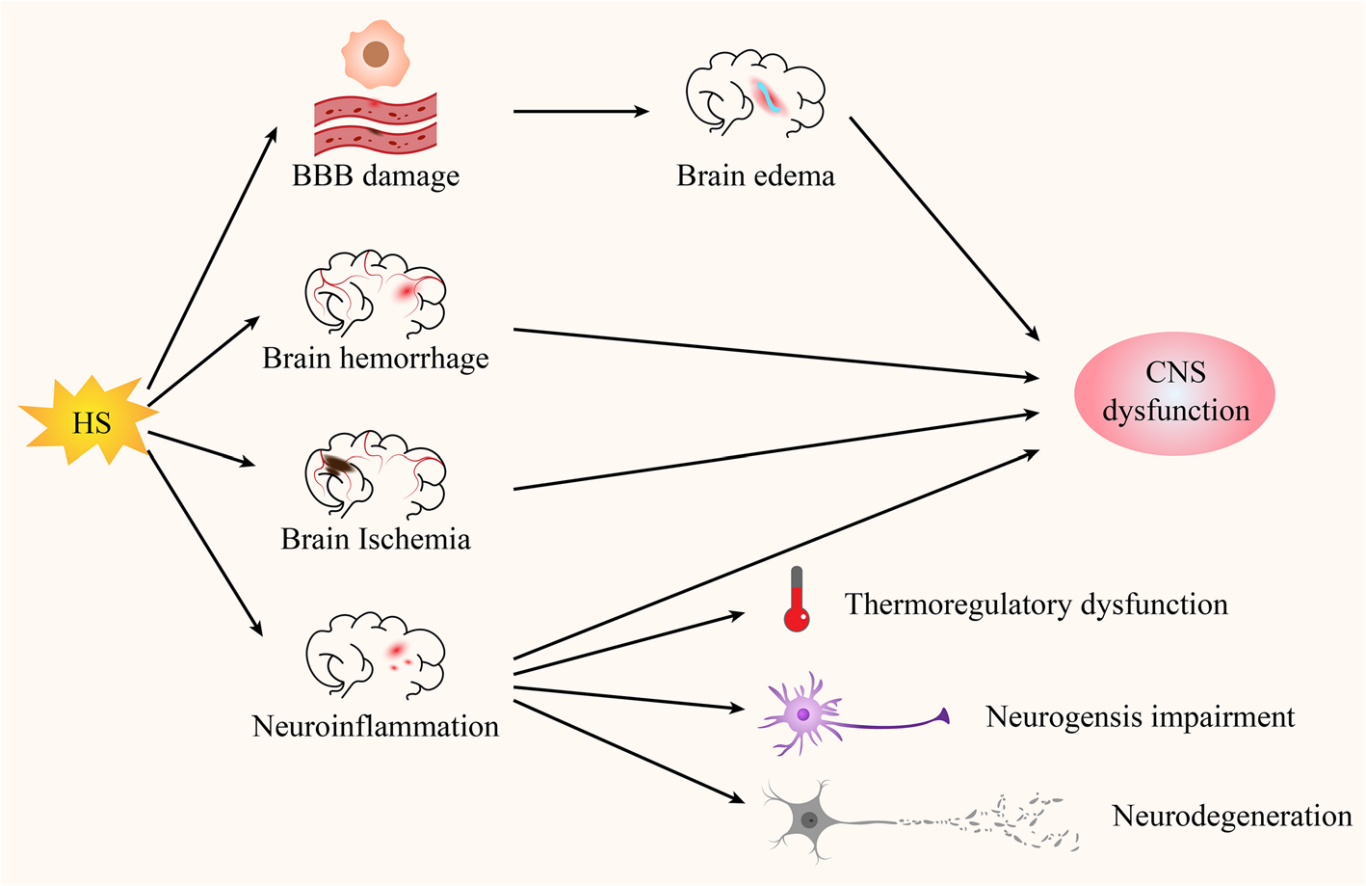
Negative effects of HS on the CNS. HS can destroy the integrity of the BBB and cause brain edema, and can induce brain hemorrhage, ischemia and neuroinflammation, which lead to CNS dysfunction. In addition, severe neuroinflammation contributes to the development of brain pathology, such as thermoregulatory dysfunction, neurogenesis impairment and neurodegeneration

To the best of our knowledge, the brain is extremely sensitive to high temperature, with both structure and function being damaged by HS (Walter and Carraretto 2016). Previous studies suggest that HS can induce brain perfusion reduction and CNS fatigue, destroy the integrity of the blood–brain barrier (BBB) and cause brain edema, leading to modifications in neuronal circuits, neurological defects, spasms and even brain atrophy (Sharma et al. 1998; Nybo 2007). Moreover, a multitude of pathological processes can be observed by magnetic resonance imaging (MRI) in the heat-stressed CNS, such as hemorrhage, edema, ischemia, and encephalitis (Zhang and Li 2014; Li et al. 2015). Based on these pathological processes, animals under HS may suffer severe CNS dysfunction including combativeness, delirium, seizures, and coma (Fig. 1) (Bouchama and Knochel 2002).

In addition, neuroinflammation caused by HS is likely a key mechanism underlying the development of brain pathology. For example, Chauhan et al. have previously indicated that the main cause of thermoregulatory dysfunction under HS conditions is associated with abnormal monoamine levels in the hypothalamus caused by neuroinflammation (Chauhan et al. 2017). Moreover, inflammation in the heat-stressed hypothalamus was found to exacerbate neurodegeneration and brain damage (Moon et al. 2017). Similarly, in the inflammatory-targeted hippocampus, HS was shown to display negative effects on adult neurogenesis and cognitive function (Lee et al. 2015; Zhu et al. 2023). More importantly, prolonged neuroinflammation can lead to various neurological disorders, including Parkinson's disease (PD), Alzheimer's disease (AD), amyotrophic lateral sclerosis (ALS) and Huntington's disease (HD) (Niranjan 2018). Hence, given the significance of preventing neuroinflammation-induced neurodegeneration, one needs to further explore the actions of appropriate neuroprotective drugs in different conditions.

## Chromium-mediated neuroprotection

Minerals, and specifically Cr, are vital nutrients for humans and animals to maintain and facilitate physical health. Although Cr is not an essential element, according to new evidence and a report released by the European Food Safety Authority, its pharmacological activity has been extensively studied (Vincent 2014, 2017). Cr exists mainly in two oxidized forms in nature: trivalent (Cr (III)) and hexavalent (Cr (VI)). While Cr (VI) is a recognized human carcinogen and has been shown to be neurotoxic (Wang et al. 2011; Singh and Chowdhuri 2017), accumulating evidence shows that Cr (III) can be used as a dietary supplement. Acting as a component of the glucose tolerance factor, Cr is important for enhancing the effect of insulin (Siddiqui et al. 2014). Therefore, given its relevance in improving carbohydrate and lipid metabolism, it is commonly used in research addressing treatments for diabetes and obesity in humans and animal models (Vincent 2001; Tian et al. 2013; Maret 2019; Wo et al. 2023). Recently, dietary Cr supplementation is becoming widely used in livestock husbandry with the aim of improving growth performance, production capacity, immune function, and antioxidative ability (Zheng et al. 2016; Sahin et al. 2017; Bin-Jumah et al. 2020; Bompadre et al. 2020; Piray and Foroutanifar 2021). Moreover, several studies are emerging on the protective effect of Cr to the brain. Dietary supplementation of Cr was shown to alleviate post-stroke brain infarction and hyperglycemia in rats (Chen et al. 2016). Oral Cr administration was found to increase concentrations of 5-hydroxytryptamine and tryptophan in the serum and brain, improving neurological function in terms of learning and memory (Orhan et al. 2017). More importantly, in depression, a condition extremely harmful to people's mental health, the administration of low-dose antidepressants supplemented with Cr was found to be an effective mitigation method (Młyniec et al. 2014; Khodavirdipour et al. 2020).

The study on the neuroprotective mechanisms of Cr has attracted the attention of many scientists because of its beneficial effects on CNS pathophysiology. Its main mechanisms of action include increased insulin sensitivity in the brain (Krikorian et al. 2010) and anti-inflammatory and antioxidant effects (Sahin et al. 2010; Akhtar et al. 2020). As a second messenger, Cr is responsible for expanding insulin signal transduction, further enhancing its role in metabolism (Vincent 2015). Brain insulin plays a vital role in regulating both systemic metabolism and brain function, being involved in feeding, depression, cognitive behavior, and energy homeostasis (Agrawal et al. 2021; Schell et al. 2021). Moreover, insulin is also involved in the maintenance of protein homeostasis, affecting the clearance of amyloid β (Aβ) peptide and the phosphorylation of tau, both known AD protein markers (Kellar and Craft 2020). Hence, when insulin resistance — defined as a reduced efficiency of insulin in promoting glucose uptake and utilization — occurs, the brain and systemic energy metabolism is disrupted and neurodegeneration may be induced (Lebovitz 2001; Sędzikowska and Szablewski 2021). It is particularly essential to supplement certain drugs in this period, namely with Cr, to increase insulin sensitivity. Additionally, Cr attenuates neuroinflammation by reducing pro-inflammatory cytokines levels such as tumor necrosis factor α (TNF-α) and interleukin (IL)-6 and enhances the ability of the antioxidant system to reduce oxidative stress (Sahin et al. 2012; Akhtar et al. 2020). However, the neuroprotective effects of Cr have not yet been fully covered. The insulin-related peptide IGF-1 also plays a role in maintaining the internal homeostasis of the brain (Song et al. 2016). Recent experimental evidence has shown that, under HS conditions, Cr supplementation can significantly increase serum concentrations of IGF-1 (Zha et al. 2009). Therefore, one may speculate that the neuroprotective effects of Cr may engage IGF-1 signaling.

## Chromium and IGF-1 

IGF-1 is an anabolic hormone that plays an important role in facilitating cell proliferation, dilating blood vessels and maintaining muscle mass and strength (Obradovic et al. 2019; Yoshida and Delafontaine 2020). IGF-1 is synthesized in the liver and enters nerve tissues through the BBB or cerebrospinal fluid in the choroid plexus (Carro and Torres-Aleman 2006). Interestingly, Cr appears to be involved in the nutritional regulation of IGF-1 levels and bioactivity. In skeletal muscle cells, Cr improves protein deposition by up-regulating mRNA levels of IGF-1 and IGF-1 receptor (IGF-1R) (Peng et al. 2010). Similarly, in pigs, increased IGF-1 levels due to Cr-supplemented diets were shown to play a role in regulating protein and fat metabolism (Wang et al. 2014). Contrastingly, in rats, maternal Cr consumption leads to decreased fetal IGF-1 concentrations, which may have negative effects on fetal protein levels and growth (Spicer et al. 1998). Moreover, Cheng et al. found that offspring of Cr-treated male mice display increased IGF-1 serum levels (Cheng et al. 2002). Despite the lack of mechanistic evidence, these results suggest a potential link between Cr and IGF-1 levels.

Given their role as growth factors, both IGF-1 and growth hormone (GH) exert neurotrophic and neuroregenerative actions (Bianchi et al. 2017). Therefore, the regulation of Cr on the biological activity of the GH-IGF-1 axis should also be noted. For instance, a Cr nanocomposite was found to enhance the mRNA expression and secretion of GH, facilitating the growth of finishing pigs (Wang et al. 2009). In contrast, in another experiment, finishing pigs treated with Cr methionine showed a decline in GH and IGF-1 levels (Tian et al. 2014). One possible explanation for these opposing differences may be the different forms of Cr supplementation. Hence, the potential link between Cr and IGF-1, supported by increased GH levels with Cr supplementation, is yet to be established and requires further research. Evidence suggests that Cr may attenuate neuroinflammation through anti-inflammatory and antioxidant effects (Akhtar et al. 2020) and the regulation of glial cells activity (Sahin et al. 2013), as does IGF-1 (Higashi et al. 2010; Labandeira-Garcia et al. 2017). Based on the effects of Cr on IGF-1 levels and IGF-1 signaling, we further discussed the mechanism of IGF-1 in suppressing neuroinflammation to explain the potential anti-neuroinflammation of Cr.

## Potential attenuating effects of IGF-1 on HS-induced neuroinflammation 

As a bioactive hormone, the expression levels and function of IGF-1 are susceptible to the effects of HS. In this regard, serum insulin, IGF-1, and glucose levels were found to be decreased in summer compared to winter in dairy cattle, possibly due to low dry matter intake and elevated negative energy balance (Hammond et al. 1990; Jonsson et al. 1997; Shehab-El-Deen et al. 2010). For animals suffering from HS, IGF-1 has protective effects on various tissues. Evidence indicates that, as the main targets of heat-stressed animals, oocytes supplemented with physiological concentrations of IGF-1 have been shown to have increased heat resistance, reduced reactive oxygen species (ROS) production and lowered rate of apoptosis (Rodrigues et al. 2016; Ascari et al. 2017; Lima et al. 2017). In all types of cells in the CNS, microglia are the main source of IGF-1 expression, and IGF-1R are preponderantly expressed in neurons and astrocytes (Labandeira-Garcia et al. 2017). When IGF-1 binds to its receptor, two major signaling pathways are activated: the mitogen-activated protein kinase (MAPK) pathway and the phosphatidyl inositol 3-kinase/protein kinase B (PI3K/AKT) pathway, both having critical effects on IGF-1-induced cell growth, survival, migration and proliferation (Yin et al. 2017). Importantly, its role as a neurotrophic hormone enables brain development and maturation, neuroplasticity and peripheral neuroregeneration (Rabinovsky 2004; Dyer et al. 2016; Zorina et al. 2023). IGF-1 maintains the integrity of the BBB, which is known to decline gradually during aging (Bake et al. 2016). In a rat model of ischemic stroke, systemic injection of IGF-1 promoted a 50% reduction in cerebral infarction size by binding to IGF-1R (De Geyter et al. 2016). In conditions of heat-stressed CNS, the disruption of IGF-1 signaling was shown to weaken the clearance of Aβ, which may mediate the development of neuroinflammation and neurodegeneration (Urban et al. 2012). On the other hand, chronic inflammation, in turn, was found to exacerbate insulin/IGF-1 signaling defects in the brain (Spielman et al. 2014). Hence, under HS conditions, IGF-1 signaling plays a critical regulatory role in the innate immune response and brain pathophysiology.

### IGF-1 and microglia-mediated neuroinflammation

Microglia, the resident immune cells in the CNS, are the first immune defense line of the CNS, being responsible for protecting CNS from damage and pathogen invasion. In the homeostatic brain tissue, microglia are essential facilitators of neuronal development and promoters of brain health through the secretion of trophic factors (Nayak et al. 2014). Microglia are also involved in synaptic pruning, which is highly related to cognitive function and the establishment of functional neural networks (Paolicelli et al. 2011). Contrarily, some evidence shows that overactivated microglia possess neurotoxic activity, causing neurodegeneration in the case of chronic neuroinflammation (Lindhout et al. 2021). According to their morphology, microglia are classified in three main types: resting ramified, activated, and amoeboid phagocytic (Ling and Wong 1993). Regardless of their morphological state, they perform monitoring functions by sensing changes in the microenvironment through their highly moving protrusions, a phenomenon more frequent in the resting ramified state (Nimmerjahn et al. 2005).

One of the most striking features of microglia is that when they receive internal signals (e.g., stress) or external signals (e.g., pathogen), they will become activated and transform from resting ramified cells into amoeboid phagocytic cells (Aloisi 2001). This activation is of multiplicity, depending on the source of stress and the type of pathology. According to the different phenotypic characteristics and functions, activated microglia can be divided into M1 and M2 polarization types. M1 microglia can release pro-inflammatory cytokines under the stimulation of lipopolysaccharide (LPS) or interferon-γ (IFN-γ), serving as the first line of defense of the innate immune system (Orihuela et al. 2016). M2 microglia stimulated by IL-4 or IL-13 can be competent in anti-inflammation and neuroprotection (Orihuela et al. 2016). Increasing evidence has been reported that, under HS conditions, the activity of M1 microglia is increased, promoting the production of pro-inflammatory mediators, including inducible nitric oxide synthase (iNOS), cyclooxygenase-2 (COX-2), TNF-α and IL-6 (Vezzani and Ruegg 2011; Estes and McAllister 2014; Lyman et al. 2014; Hsuan et al. 2016). Moreover, Weninger et al. found that, in hippocampal slices treated with a heat shock, the number of microglia significantly increased, based on Iba-1 positive staining, when compared with the control group, with an abundant number of actively phagocytic microglia being observed (Weninger et al. 2021). Similar results were reported in the hypothalamus during acute HS (Belity et al. 2022). Therefore, HS can induce the activation and proliferation of microglia, which may trigger neuroinflammation. Accordingly, an in-depth understanding of changes in the microglial state is of great significance for neuroinflammation.

**Fig. 2:**
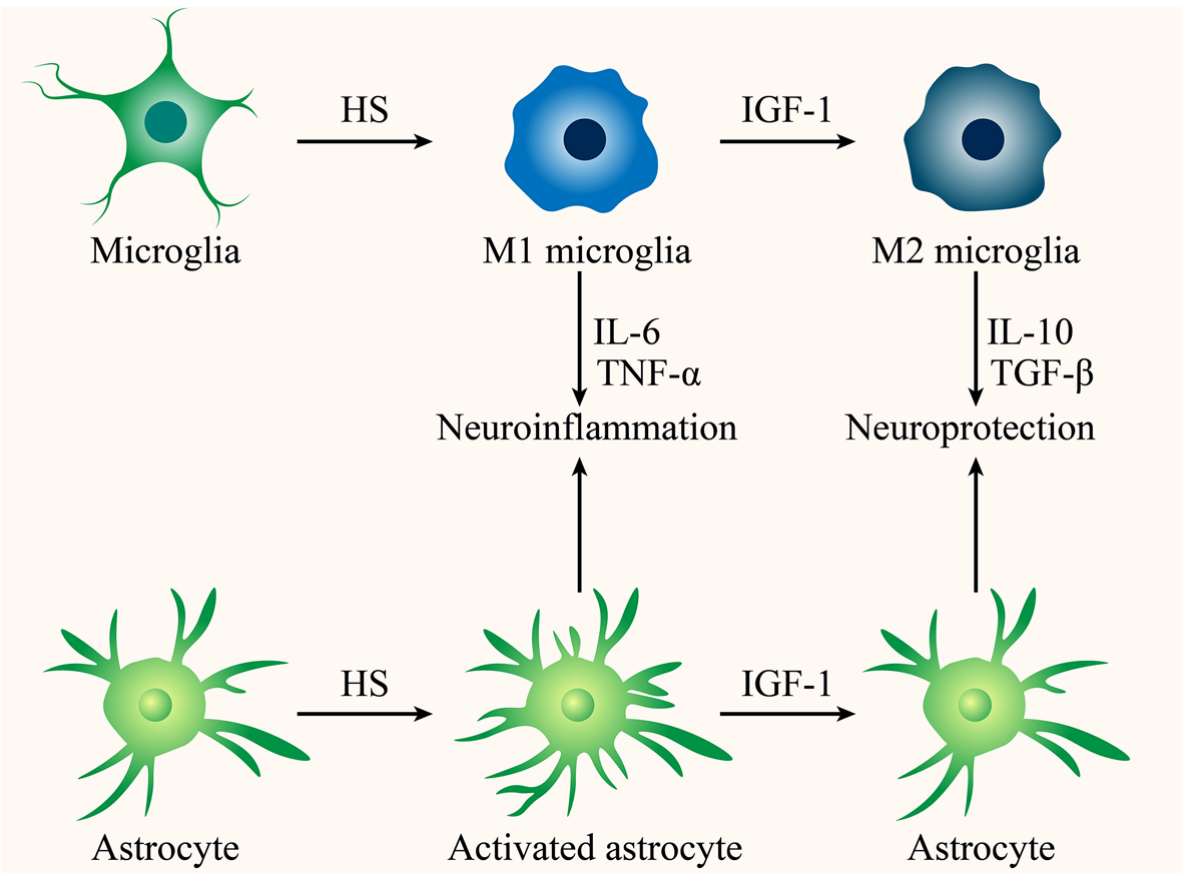
IGF-1 attenuates microglial and astrocytes-mediated neuroinflammation

Activation of microglia is the first and most critical step in neuroinflammation. IGF-1 is a mitogen for microglia that can also function as a regulator of microglial polarization to modulate microglia-mediated neuroinflammation (Fig. 2) (Labandeira-Garcia et al. 2017). In addition, IGF-1/IGF-1R signaling transduction is vital for regulating microglia morphology and transcriptome, which reduces the severity of inflammatory response (Ivan et al. 2023). As a marker of the M2 phenotype, IGF-1 was shown to promote CD206 expression in mice with intracerebral hemorrhage, accompanied by elevated levels of anti-inflammation mediators such as IL-10 and transforming growth factor β (TGF-β) (Sun et al. 2020). The expression levels of arginase-1, another enzyme associated with an anti-inflammatory microglia phenotype, were found to be increased after IGF-1 overexpression (Falomir-Lockhart et al. 2022). In addition, novel insights into the regulation of mitochondrial metabolism and autophagy on microglial polarization have emerged (Orihuela et al. 2016; Jiang et al. 2020). Indeed, Ji et al. confirmed that IGF-1 facilitated a M1-to-M2 shift of microglia by enhancing autophagy (Ji et al. 2018). Ferger et al. suggested that mitochondrial dysfunction inhibited a M2 microglia phenotype induced by IL-4 (Ferger et al. 2010). Hence, according to these pieces of evidence, perhaps IGF-1 is promoting the transformation of microglia to a M2 phenotype to alleviate neuroinflammation through the improvement of mitochondrial function (Sadaba et al. 2016; Yang et al. 2021). Furthermore, IGF-1 exhibited anti-inflammatory effects by reducing LPS-induced expression of brain inflammatory factors through the downregulation of microglia activation and production of endogenous growth factors (Sukhanov et al. 2007; Park et al. 2011a, b; Tien et al. 2017). In senile rats (Falomir-Lockhart et al. 2019) and traumatic intracerebral hemorrhage models (Herrera et al. 2021), IGF-1 gene therapy was also found to modulate the proliferation of reactive microglia. Taken together, multiple pieces of evidence indicates that a strict regulation of IGF-1 levels is essential for the modulation of microglial inflammatory responses in the CNS, which provides a reference for exploring the regulation of Cr on the activity of microglia.

### IGF-1 and astrocytes-mediated neuroinflammation

Astrocytes are abundant glial cells in the brain tissue and perform complex and diverse functions, including nutrient supply to neurons, synaptic integrity maintenance, and regulation of local CNS blood volume (Ransom et al. 2003). Astrocytes become activated in response to various adverse stimuli, a process defined by increased expression of glial fibrillary acidic protein (GFAP) (Sofroniew 2009). After activation, astrocytes release pro-inflammatory signaling molecules, particularly in the cortex and midbrain, in order to build up responses to innate immune triggers (Kipp et al. 2008). In addition to their pro-inflammatory role, it has been demonstrated that cytotoxic molecules secreted by astrocytes greatly contribute to neurodegenerative pathophysiology, especially in AD (Liddelow et al. 2017; Li et al. 2019a, b). Another way in which astrocytes respond to various forms of CNS damage is the double-edged sword process of reactive astrogliosis. On the one hand, glial scars formed during severe reactive astrogliosis have neuroprotective effects that limit the spread of inflammation and pathogens (Sofroniew and Vinters 2010). On the other hand, loss of physiological function or gain of adverse effects of reactive astrocytes may be the pathological basis of CNS dysfunction, such as trauma and stroke (Sofroniew 2009). Accumulating evidence indicates that acute HS can induce increased expression of reactive astrocytes actively involved in different pathophysiological processes in a short period of time (Sharma et al. 1992; Moon et al. 2017). In fact, frequent repetitive thermal stimulation to the hippocampus was shown to prolong the activation time of astrocytes concomitant with increased expression of GFAP (Yang et al. 2009). In addition, Chauhan et al. discovered that severe HS promotes an increased expression of GFAP in the hypothalamus accompanied by inflammation (Chauhan et al. 2017). In conclusion, astrocytes can also become activated and proliferate, contributing to the secretion of pro-inflammatory cytokines and chemokines that induce inflammation (Banjara and Ghosh 2017).

Evidence has shown that IGF-1 plays a vital role in regulating astrocytic activity (Fig. 2) (Labandeira-Garcia et al. 2017). According to Fernandez et al., further investigation on the regulation of astrocyte function by IGF-1 is essential to fully understanding the biological role of IGF-1 in the brain (Fernandez et al. 2007). The modulation mediated by IGF-1 on mitochondrial dynamics and the redox state of astrocytes is important for astrocyte function (Logan et al. 2018). IGF-1 regulates the energy supply of the brain by promoting astrocytic glucose uptake (Hernandez-Garzón et al. 2016). Severely, mice lacking IGF-1R in astrocytes show cognitive impairment, which is related to the development of the AD-like pathology (Zegarra-Valdivia et al. 2022). In addition, IGF-1 targeting astrocytes-mediated neuroinflammation is a crucial part of its neuroprotective effects. When astrocytes are exposed to IGF-1 for a long time, sustained activation of IGF-1R will inhibit astrocytic mitosis through the upregulation of phosphatase and tensin homolog deleted on chromosome ten (PTEN) activity (Fernandez et al. 2007). Accumulating reports also show that IGF-1 is able to reduce the number of GFAP-positive astrocytes in aging and nerve injury models (Miltiadous et al. 2011, Park et al. 2011a, b, Arroba and Valverde Á 2015, Okoreeh et al. 2017), which is similar with a Cr-induced GFAP fall in hypoglycemic rats (Sahin et al. 2013). In line with this, reactive astrocytes play an active anti-inflammatory role under the actions of IGF-1, suppressing the expression of pro-inflammatory cytokines and enhancing the secretion of neuroprotective factors (Fernandez et al. 2007). Recent experimental evidence indicates that an IGF-1 gene therapy effectively controlled reactive astrogliosis and attenuated astrocyte-mediated neuroinflammation induced by LPS (Bellini et al. 2011; Pardo et al. 2016). Nonetheless, given the two opposing facets of reactive astrogliosis, further discussion is needed on the role of IGF-1 in the regulation of astrocytic activity.

### IGF-1 inhibits signaling pathways involved in neuroinflammation

In the CNS, heat shock proteins (Hsps) are highly conserved proteins synthesized by glial cells in response to HS (Taylor et al. 2007). Hsps act as molecular chaperones that promote the proper folding of nascent polypeptides and the repair of damaged proteins, playing a vital role in maintaining protein homeostasis. Oligodendrocytes are myelin-forming cells in the CNS that have been shown to provide nutrients to neurons (Kipp 2020). Nevertheless, under HS conditions, oligodendrocytes seem to play a neuroinflammatory role. Pavlik et al. found that oligodendrocytes, but not microglia and astrocytes, are major producers of Hsp70, the most ubiquitous and conserved Hsp, in rats subjected to heat-stressed conditions with circulating hot air (41.5℃) (Pavlik et al. 2003). Likewise, several reports have revealed that HS can induce increased expression of Hsp70 in the brain tissue (Moon et al. 2017; Kim et al. 2019). When extracellular Hsp70 binds to its receptor, a specific inflammatory signaling pathway is initiated (Dukay et al. 2019). Indeed, Hsp70 is an ideal damage-associated molecular pattern (DAMP) recognized by Toll-like receptor 4 (TLR4) that is highly conserved and significantly increased after HS (Dukay et al. 2019). TLR4 is mainly expressed on the membrane of microglia and astrocytes in nerve tissues, being a key receptor that contributes to the induction of inflammation and antiviral cytokines (Kumar 2019; Li et al. 2019a, b). After the binding of extracellular Hsp70 to TLR4, activated TLR4 induces the recruitment of downstream adaptors that lead to the activation of nuclear factor-kappa B (NF-κB) (Kawai and Akira 2010). Next, a large number of pro-inflammatory mediators are produced that accelerate neuroinflammation development (Kagan and Medzhitov 2006; Kim and Yenari 2013; Banjara and Ghosh 2017). Hence, there is not only a close relationship between the regulation of inflammatory signaling with Hsp70 expression but also between HS-induced neuroinflammation and the activation of the TLR4/NF-κB pathway.

Elevated Hsp70 expression is thought to reflect the response of nerve cells to stress and injury, which can eventually lead to neuroinflammation (Lee et al. 2000; Akbar et al. 2001; Giuliano et al. 2011). Kazanis et al. found that IGF-1 suppresses Hsp70 expression in the brain injury area, contributing to the alleviation of neuronal degeneration and death (Kazanis et al. 2003). Similar results were reported in the neurotoxic substance-induced hippocampal degeneration model (Miltiadous et al. 2010). Therefore, IGF-1 attenuates tissue overreaction to injury by reducing Hsp70 production. Notably, the anti-inflammatory effect of IGF-1 is known to be related to the inhibition of the TLR4/NF-κB pathway. In pathological processes such as lung injury (Munoz et al. 2021), cirrhosis (Zhao et al. 2021a, b), and enteritis (Tian et al. 2017), it has been demonstrated that IGF-1 inhibits the activation of the TLR4/NF-κB pathway, impeding cell damage and enhancing tissue repair. A study by Lee and colleagues indicated that the negative regulation of IGF-1 on TLR4 expression is highly associated with the PI3K/AKT pathway and the decreased expression of many NF-κB-mediated pro-inflammatory factors (e.g., TNF-α, IL-6) (Lee 2011). Moreover, recent evidence confirmed that IGF-1 has the ability to downregulate the expression of TLR4 and to mediate the inactivation of NF-κB by activating the PI3K/AKT pathway to reduce the astrocytic inflammatory response (Pinto-Benito et al. 2022). Moreover, IGF-1 can promote M2 microglial polarization via the inhibition of TLR4/NF-κB signaling transduction and lead to the elevation of neuroprotection factors such as IL-10 and TGF-β to attenuate microglia-mediated inflammation (Sun et al. 2020). Importantly, the reduced Hsp70 production and the inhibition of TLR4/ NF-κB pathway can be achieve by Cr supplement (Zhang et al. 2014; Kumar et al. 2015). The inhibitory effects of Cr and IGF-1 on inflammatory signaling pathways greatly facilitate the control or resolution of inflammation, providing compelling perspectives on the neuroprotective role of IGF-1 and the potential between Cr and IGF-1.

### IGF-1 and oxidative stress

Mitochondria are not only the energy production center of cells but also the center of pro-inflammatory responses (Andrieux et al. 2021). HS is a known inducer of mitochondrial dysfunction in rat CNS neurons and can lead to a decreased ability to neutralize or to the overproduction of ROS (White et al. 2012). Additionally, NADPH oxidases (NOX) like NOX-1 and NOX-4 are involved in ROS production under HS conditions (Moon et al. 2010; Kikusato et al. 2015). To prevent cells from oxidative damage, mitochondria have an antioxidative defense capability. However, HS inhibits the total antioxidant capacity (T-AOC), which is characterized by the presence of glutathione and enzymatic antioxidants such as superoxide dismutase (SOD), catalase (CAT), glutathione peroxidase (GPx) and hemeoxygenase-1 (HO-1) (Liu et al. 2016; Wang et al. 2019; Chauhan et al. 2021; Oghbaei et al. 2021). Moreover, the concentration of malondialdehyde (MDA), an indicator of lipid peroxidation, has been shown to be increased in the diencephalon of chicks exposed to heat (Chowdhury et al. 2014). Collectively, HS induces the excessive production of ROS and reduces the antioxidative capacity of the system, resulting in oxidative damage. Oxidative stress is a crucial potential factor of inflammatory responses. ROS accelerate the recruitment of inflammatory cells and increase the expression of cytokines and chemokines by activating different transcription factors, including NF-κB (Guzik et al. 2003; Kim et al. 2013; Ding et al. 2020; Eckert et al. 2021). In the brain tissue, ROS induce inflammation and neuron death, which in turn mediates neurodegeneration and memory loss (Popa-Wagner et al. 2013).

Nuclear factor erythroid 2-related factor 2 (Nrf2) is a sensor and transcriptional mediator of antioxidants and oxidative signals, being a great preventor of oxidative damage (Ma et al. 2006). After the activation of Nrf2, it can be transferred into the nucleus, where it binds to the antioxidative response element (ARE), initiating a downstream pathway for signal transduction that promotes the synthesis of enzymatic antioxidants, including SOD, CAT, and HO-1 (Jung and Kwak 2010). Recent experimental evidence has shown that increased levels of IGF-1 prevent renal cells against oxidative damage induced by cisplatin (Mahran 2020). In addition, IGF-1/IGF-1R signaling in the brain was found to activate the Nrf2/HO-1 pathway, an important antioxidant and anti-inflammatory pathway (Kim et al. 2012; Luo et al. 2017; Ma et al. 2020; Niu et al. 2020), and Cr plays an antioxidative role through enhancing the Nrf2 activity and the antioxidants expression levels (Sahin et al. 2017; Chen et al. 2021a, b). According to Wang et al., IGF-1 protects SH-SY5Y cells against Aβ-induced cell injury by reducing ROS through the PI3K/Akt-Nrf2 signaling pathway in an AD model (Wang et al. 2017). Similarly, Sui et al. reported that IGF-1 improved tau pathology induced by high-fat diet via the activation of the Nrf2/HO-1 signaling pathway (Sui et al. 2021). Moreover, knockdown of IGF-1 was shown to reduce antioxidant defenses by impairing the Nrf2-dependent antioxidant response (Bailey-Downs et al. 2012). Collectively, Nrf2/HO-1 is an important antioxidative pathway that mediates Cr and IGF-1 actions. Furthermore, increasing evidence now suggests that low doses of IGF-1's can effectively ameliorate mitochondrial dysfunction, resulting in reduced ROS production, oxidative damage and apoptosis, and in an elevation of ATP production (Puche et al. 2008; Sadaba et al. 2016; Yang et al. 2021; Lv et al. 2022). These conclusions suggest that IGF-1 can ameliorate mitochondrial dysfunction and enhance the function of the antioxidant defense system to neutralize or scavenge the excessive production of ROS induced by HS.

### IGF-1 and endoplasmic reticulum stress

Endoplasmic reticulum (ER) is an important organelle responsible for protein synthesis in cells and plays a critical role in assisting protein modification and folding (Gong et al. 2017). Aggregation of unfolded or misfolded proteins in ER under adverse conditions leads to ER stress. Signal transduction of unfolded proteins depends on three resident transmembrane proteins on the endoplasmic reticulum membrane: inositol-requiring enzyme 1α (IRE1α), pancreatic endoplasmic reticulum kinase (PERK), and activating transcription factor 6 (ATF6) (Chandrika et al. 2015; Oakes and Papa 2015). As classical markers of ER stress, glucose-regulated protein 78 (GRP78) and C/EBP-homologous protein (CHOP) expression levels are increased under HS conditions (Dong et al. 2017). Furthermore, excessive production of ROS may disrupt ER homeostasis by aggravating oxidative damage (Zhang et al. 2016). Compelling evidence now demonstrates that ER stress can induce the activation of microglia, astrocytes and NF-κB, representing a key step in the development of neuroinflammation (Meares et al. 2014; Harvey et al. 2015; Sprenkle et al. 2017). Other lines of evidence indicate that HS increases the levels of misfolded proteins, resulting in ER-triggered apoptosis (Chen et al. 2008; Nasrolahi et al. 2020). Moreover, ER stress was found to inhibit the transcription of the IGF-1 gene, resulting in decreased IGF-1 sera and brain tissue levels (Marwarha et al. 2016; Xia et al. 2020). However, IGF-1 seems to be a new target for reducing ER stress in the CNS. Recent experimental results showed that in fibroblasts the increased expression levels of IGF-1 inhibited the expression of ER stress-related genes (Di Patria et al. 2022). According to Fang et al., under high glucose conditions, IGF-1 alleviates ER stress and ER stress-induced apoptosis in rat gastric smooth muscle cells (Fang et al. 2019). Furthermore, IGF-1 was found to enhance the expression of CHOP and to inhibit the phosphorylation of eIF2α, thus attenuating 6-OHDA-induced ER stress-mediated apoptosis in PC-12 neuronal cells (Kim et al. 2012). Before that, Zou et al. reported that IGF-1 effectively protected PC-12 neuronal cells against ER stress-induced apoptosis through the PI3K/Akt and p38 MAPK pathways(Zou et al. 2009). In addition, gestational diabetes rats with Cr supplement showed that the reduced GRP78 level and IRE1α activity can prevent liver from ER stress (Yao et al. 2021). Hence, the abovementioned evidence suggests that Cr and IGF-1 play positive roles in inhibiting HS-induced ER stress, which provides prospective about Cr promoting IGF-1 actions.

## Conclusion

**Fig. 3:**
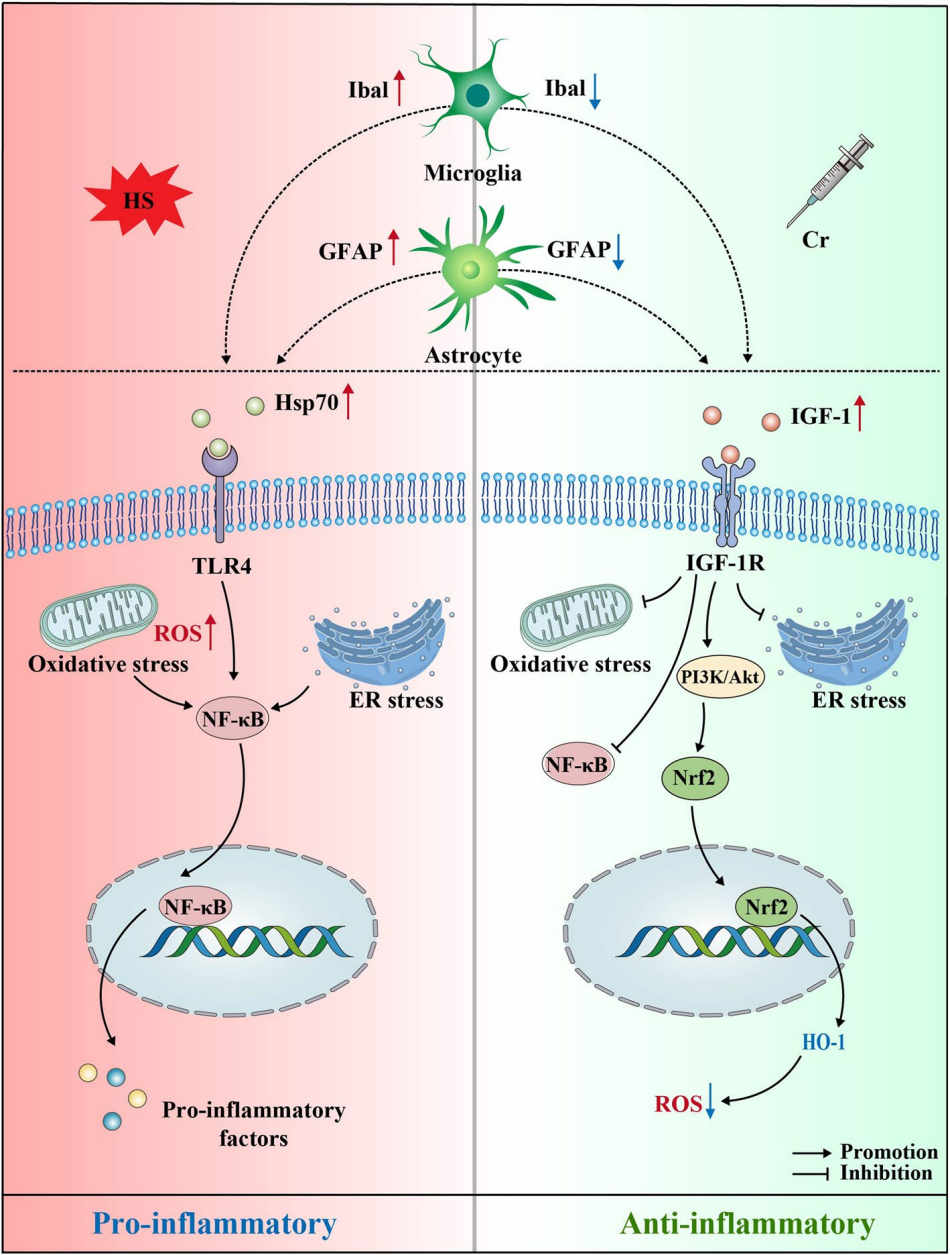
Cr attenuates HS-induced neuroinflammation via the neuroprotective effects of IGF-1. HS induces the proliferation of microglia and astrocytes, which can be inhibited by Cr-induced IGF-1. Under HS conditions, when Hsp70 binds to TLR4, the NF-κB is activated to facilitate the expression of pro-inflammatory factors. This can also happen after oxidative stress and ER stress, leading to neuroinflammation. Cr supplementation can increase IGF-1 levels which, in turn, suppress the expression of Hsp70. After the binding of IGF-1 to IGF-1R, the PI3K/Akt pathway is activated, which inhibits the activation of the TLR4/NF-κB signaling transduction to reduce the expression of pro-inflammatory factors. Moreover, IGF-1 enhances antioxidative defense via the PI3K/Akt-Nrf2/HO-1 pathway to clear the excessive production of ROS. Additionally, through PI3K/Akt pathway, IGF-1 ameliorates ER stress to prevent the apoptosis of neuronal cells

With global warming, the frequency of HS increases, and its adverse effects on the CNS cannot be ignored. As demonstrated in this present review, HS can activate glial cells and the NF-κB pathway to facilitate the production of pro-inflammatory cytokines that lead to neuroinflammation. Moreover, HS-induced oxidative stress and ER stress further contribute to the development of neuroinflammation, cell death and tissue damage. If inflammation becomes unmanageable, it may lead to several neurological diseases that greatly threaten and harm the normal lives of animals. Cr, an important mineral present in nature, has been shown to play an important anti-inflammatory and antioxidant role in the brain. Interestingly, elevated production of IGF-1 is induced by the supplementation with Cr. As a neuroprotective factor, IGF-1 mediates the anti-inflammatory effects of microglia and astrocytes. In addition, IGF-1 decreases Hsp70 levels and inhibits the activation of the TLR4/NF-κB pathway to suppress the expression of pro-inflammatory cytokines. Moreover, IGF-1 attenuates oxidative stress and ER stress via the PI3K/Akt pathway to ameliorate cell damage. Therefore, one can conclude that Cr efficiently ameliorates neuroinflammation evoked by HS through the mediation of IGF-1 signaling (Fig. 3). Considering the putative pro-inflammatory effects of IGF-1 in other backgrounds, further experiments focused on HS are needed to verify this hypothesis. The findings of these upcoming studies will create the rationale for an effective approach in the prevention and treatment of neurological diseases associated with HS.

## Data Availability

Not applicable.

## Abbreviations

ATF6: Activating transcription factor 6
AD: Alzheimer's disease
Aβ: Amyloid β
BBB: Blood-brain barrier
CHOP: C/EBP-homologous protein
CAT: Catalase
CNS: Central nervous system
Cr: Chromium
COX: Cyclooxygenase
DAMP: Damage-associated molecular pattern
ER: Endoplasmic reticulum
GFAP: Glial fibrillary acidic protein
GRP78: Glucose-regulated protein 78
GPx: Glutathione peroxidase
GH: Growth hormone
Hsp70: Heat shock protein 70
HS: Heat stress
HO-1: Hemeoxygenase-1
iNOS: Inducible nitric oxide synthase
IRE1α: Inositol-requiring enzyme 1α
IGF-1: Insulin-like growth factor 1
IGF-1R: Insulin-like growth factor 1 receptor
IFN: Interferon
IL: Interleukin
LPS: Lipopolysaccharide
MDA: Malondialdehyde
MAPK: Mitogen-activated protein kinase
Nrf2: Nuclear factor erythroid 2-related factor 2
NF-κB: Nuclear factor-kappa B
PEPK: Pancreatic endoplasmic reticulum kinase
PI3K: Phosphatidyl inositol 3-kinase
AKT: Protein kinase B
ROS: Reactive oxygen species
SOD: Superoxide dismutase
TLR4: Toll-like receptor
T-AOC: Total antioxidant capacity
TGF: Transforming growth factor
TNF: Tumor necrosis factor
